# Longitudinal Prediction of Freezing of Gait in Parkinson's Disease: A Prospective Cohort Study

**DOI:** 10.3389/fneur.2021.758580

**Published:** 2022-01-03

**Authors:** Jiahao Zhao, Ying Wan, Lu Song, Na Wu, Zien Zhang, Zhenguo Liu, Jing Gan

**Affiliations:** Department of Neurology, Xinhua Hospital Affiliated to Shanghai Jiao Tong University School of Medicine, Shanghai, China

**Keywords:** Parkinson's disease, freezing of gait, prediction model, nomogram, longitudinal

## Abstract

**Objective:** Freezing of gait (FOG) is a disabling complication in Parkinson's disease (PD). Yet, studies on a validated model for the onset of FOG based on longitudinal observation are absent. This study aims to develop a risk prediction model to predict the probability of future onset of FOG from a multicenter cohort of Chinese patients with PD.

**Methods:** A total of 350 patients with PD without FOG were prospectively monitored for ~2 years. Demographic and clinical data were investigated. The multivariable logistic regression analysis was conducted to develop a risk prediction model for FOG.

**Results:** Overall, FOG was observed in 132 patients (37.70%) during the study period. At baseline, longer disease duration [odds ratio (OR) = 1.214, *p* = 0.008], higher total levodopa equivalent daily dose (LEDD) (OR = 1.440, *p* < 0.001), and higher severity of depressive symptoms (OR = 1.907, *p* = 0.028) were the strongest predictors of future onset of FOG in the final multivariable model. The model performed well in the development dataset (with a C-statistic = 0.820, 95% CI: 0.771–0.865), showed acceptable discrimination and calibration in internal validation, and remained stable in 5-fold cross-validation.

**Conclusion:** A new prediction model that quantifies the risk of future onset of FOG has been developed. It is based on clinical variables that are readily available in clinical practice and could serve as a small tool for risk counseling.

## Introduction

Freezing of gait (FOG) is a dramatic gait difficulty defined as “a brief, episodic absence or marked reduction of forward progression of the feet despite the intention to walk (1).” It is a disabling symptom that increases the probability of falls, fractures and contributes to immobility, loss of independence, reducing the quality of life of patients with Parkinson's disease (PD) (2). However, at the very early stage of FOG, the physician seldomly observed it in the consulting room because of the episodic nature of FOG. Without targeted questioning, both patients and physicians were prone to overlooking FOG (3). Therefore, early identification of individuals at risk of developing FOG could help to stratify the PD population for clinical trials, counsel individual patients on their prognosis, and enable clinicians to better manage the disease throughout.

Several longitudinal follow-up studies reported risk factors of future FOG onset including demographic parameters, motor symptoms, non-motor symptoms, laboratory parameters, neuroimaging data, and medication status (4–12). However, these risk factors were rarely used to predict the onset of FOG in clinical practice (13). Most prediction models were derived from small, single-center development datasets; therefore, representativeness and credibility are limited. A FOG risk model is an example of a prognostic model (13). Such models should ideally be developed by taking a large cohort of patients with PD without FOG, measuring baseline risk factors, and following the cohort for a sufficiently long time to see who develops FOG (14). This study aimed to develop a risk prediction model incorporating various elements available through clinical investigations to predict the probability of future onset of FOG in patients with PD. Such a model would be a useful tool for clinicians diagnosing FOG and making therapeutic decisions.

## Materials and Methods

### Study Design, Populations, and Procedures

Model development was performed using data based on a subset of patients with PD derived from a multicenter, cross-sectional study (clinical registration No: NCT03026595) between October 2017 and November 2018. The total cohort included 411 patients with PD who agreed to be followed for ~2 years (range from 20 to 29 months) among 10 hospitals in China. Ethical approval was obtained from the Research Ethics Committee of each study center. All the participants provided a written informed consent. Clinical diagnoses of PD were based on the criteria from the UK Brain Bank. The diagnosis was revaluated by a movement disorder specialist at 2 years of follow-up. The exclusion criteria included serious comorbidities likely to affect gait performance, e.g., stroke, trauma or orthopedic disease, and severe internal diseases. Patients with brain surgery including deep brain stimulation (DBS) were also excluded. A face-to-face visit was administrated at baseline. Details with regard to demographics, comorbidities, and medication usage were obtained. All the patients were scheduled for a 2-year telephone follow-up at first. Then, patients who were unsure about their onset of FOG symptoms were arranged for a face-to-face visit to determine, if FOG has occurred (103 out of 411 participants eventually). More severely affected patients were offered the possibility to be examined at their homes to minimize selective dropout.

### Outcome Measure

The presence or absence of FOG was assessed via a self-report structured questionnaire, the New FOG Questionnaire (NFOG-Q) item 1 (15). FOG was also identified when it was observed by an experienced clinician during a visit or it was reported by the patients, their family members, or their caregivers when it occurred at home or anywhere outside of the hospital. Participants were asked to walk in a narrow passage, pass through a narrow doorway, make full and rapid turns, and walk under dual task to see, if FOG could be provoked in the clinic during a visit. The typical FOG phenomenon was demonstrated to the patients, their family members, or their caregivers by an experienced clinician to help them recognize the onset of FOG.

### Candidate Predictors

We built the model using a limited number of candidate variables, which were preselected based on clinical expertise, scientific evidence, and practical feasibility. In a recently published study, we identified relevant risk factors for FOG development in PD (16). We searched for predictors of FOG that were repeatedly reported in studies without language or time restrictions. Eligible studies had a primary endpoint of the presence of FOG assessed with the Movement Disorder Society-Unified PD Rating Scale (MDS-UPDRS) item 2.13, the UPDRS II item 14, the FOG-Q item 3, the NFOG-Q item 1, or objective observation (6, 7, 11, 12, 17). Based on these results of published studies, we selected 12 predictor variables in the prediction models including age, sex, modified Hoehn and Yahr (mH&Y) stage, disease duration, and following clinical characteristics. Motor impairment was assessed with the UPDRS-motor examination (UPDRS-ME) section. The tremor dominant (TD) score and postural instability and gait difficulty (PIGD) score were calculated using established methods (18). Motor fluctuations were defined as the presence of predictable wearing-off or unpredictable off periods (UPDRS item 36, 37). Total levodopa equivalent daily dose (LEDD) was calculated according to the previously suggested conversion formula (19). The Berg Balance Scale (BBS) was used to evaluate the balance of patients with PD. The presence of anxious and depressive symptoms was assessed with the Hamilton Anxiety Rating scale (HAMA) and the Hamilton Depression Rating Scale (HAMD) (24 items). The severity of depressive symptoms was evaluated using the HAMD (24 items), with a score of < 8 indicating no depression, a score from 8 to 20 corresponding to mild depression, a score from 20 to 35 corresponding to moderate depression, and a score of > 35 corresponding to severe depression (20). Global cognitive function was assessed by the Montreal Cognitive Assessment (MoCA). Considering that the aim of this study was to develop a simple and easy-to-use model, cerebrospinal fluid parameters limited by accessibility were excluded in this study.

### Sample Size

The sample size estimate is based on a rule of thumb for developing risk prediction models with unbiased estimates of regression coefficients (21). We calculated the sample size needed for the development of the model based on the need for 10–15 new-onset patients with FOG per risk factor plus 10% dropout. We assumed the proportion of patients with PD presenting with FOG at 2 years to be between 30 and 40%, based on the previously reported longitudinal Chinese population-based cohorts (6, 7, 12). The sample size needs to be at least 333–444 to develop a reliable model with minimal overfitting with 12 candidate predictor variables. Conversion rates varied significantly among studies, especially follow-up intervals were not uniform. Because it was not possible to estimate the precise event rate, we tried to put the group size as close as possible to 444 people during the recruitment as a more conservative 30% event rate.

### Model Development

Based on the NFOG-Q scores at follow-up, patients with PD who did not suffer from FOG at both timepoints were classified as the non-FOG group and patients who “converted” and became freezers during the 2 years follow-up were classified as conversion into the FOG group. We developed prediction models using binary logistic regression to predict the occurrence of FOG. Multivariable regressions including 12 candidate predictor variables were performed with a stepwise backward selection strategy. We used the stepAIC function in the Modern Applied Statistics with S (MASS) package in R (22) for stepwise regression models based on the exact Akaike information criterion (AIC) criterion. We used the bootstrap approach to generate the training dataset by drawn with replacement from the original dataset (resample 1,000 times stratified by FOG development to preserve the conversion rate of original dataset). For each bootstrap replication, a stepwise backward selection method is used to identify the significant variables. Candidate predictors that appeared in more than 50% of the multivariable models generated in the different bootstrap samples were retained in the final model. Regression coefficients and SEs of the final model were averaged using Rubin's rules (23). The assumption of linearity in the logit for the continuous independent and dependent variables was assessed by the Box–Tidwell test. Multicollinearity was assessed using the Pearson's correlation coefficient statistic. Then, the variance inflation factor (VIF) was calculated based on a multiple regression model incorporating the same dependent and independent variables. Multicollinearity was considered to exist if r ≥ 0.40 or VIF ≥ 2.5.

### Model Performance and Validation

The discriminative ability assesses the model differentiation capability whether the patients develop FOG in the future. It was evaluated with the area under the receiver operator characteristics curves (C-statistic) (24). Model calibration assesses to what extent predicted values agree with observed outcomes. It is visualized by the calibration plot in which the calibration curve is estimated by locally estimated scatterplot smoothing (LOESS) (24). It represents the average predictor effects. The calibration slope has an ideal value of 1. If the value is <1, it means that the model is overfitting, implying higher model complexity than the actual problem and poor generalization ability. If the value is >1, it means that the model is underfitting, implying low model complexity and the model performs poorly on the dataset. The calibration intercept has an ideal value of 0 (25). Overall agreement between predicted and observed outcomes is tested using the Hosmer–Lemeshow goodness of fit test.

Apparent model performance estimated directly from bootstrap sample that was used to develop prediction model. Since these parameters are generated in 1,000 bootstrap samples, for each parameter the median is shown. The final model was validated by internal validation and we used the bootstrap approach to validate the final model. The original dataset was resampled 1,000 times with replacement, in which regression models were fitted in these bootstrap replicates. The model development process was repeated in each bootstrap sample (including variable selection) to produce a model. Each model was applied to the same bootstrap sample to quantify apparent performance and also to the original dataset to test model performance (C-statistic, calibration slope, and intercept) and optimism (difference in test performance and apparent performance). The optimism-corrected calibration C-statistic, calibration slope, and intercept were estimated to evaluate the stability of a prediction model to random changes in sample composition.

Since several sites had insufficient FOG events required for reliable internal–external cross-validation, we use both bootstrap and 5-fold cross-validation to complete the internal validation process. Internal validation could check the repeatability of the model development process and prevent overfitting of the model leading to overestimation of the model performance. The total cohort is divided into five subsets and the holdout method is repeated five times. Each time, one of the 5 subsets is used as the test set and the other four subsets are put together to form the training set.

Missing data were only found for one variable, namely MoCA scores in five participants. Missing values were imputed with median using the simple imputation method for final calculations. The distribution of the MoCA score did not show significant changes after imputation. We compared groups using the chi-squared tests, the *t*-tests for normally distributed variables, and the Mann–Whitney *U* tests for non-parametric data. For all the statistical tests, we used 0.05 as the significance level. Statistical uncertainties were expressed using 95% CIs. Statistical analyses were performed with R version 4.0.3 (R Foundation for Statistical Computing, Vienna, Austria; http://www.R-project.org/). This study is reported in compliance with the Transparent reporting of a multivariable prediction model for individual prognosis or diagnosis (TRIPOD) statement (26).

## Results

**Figure 1:**
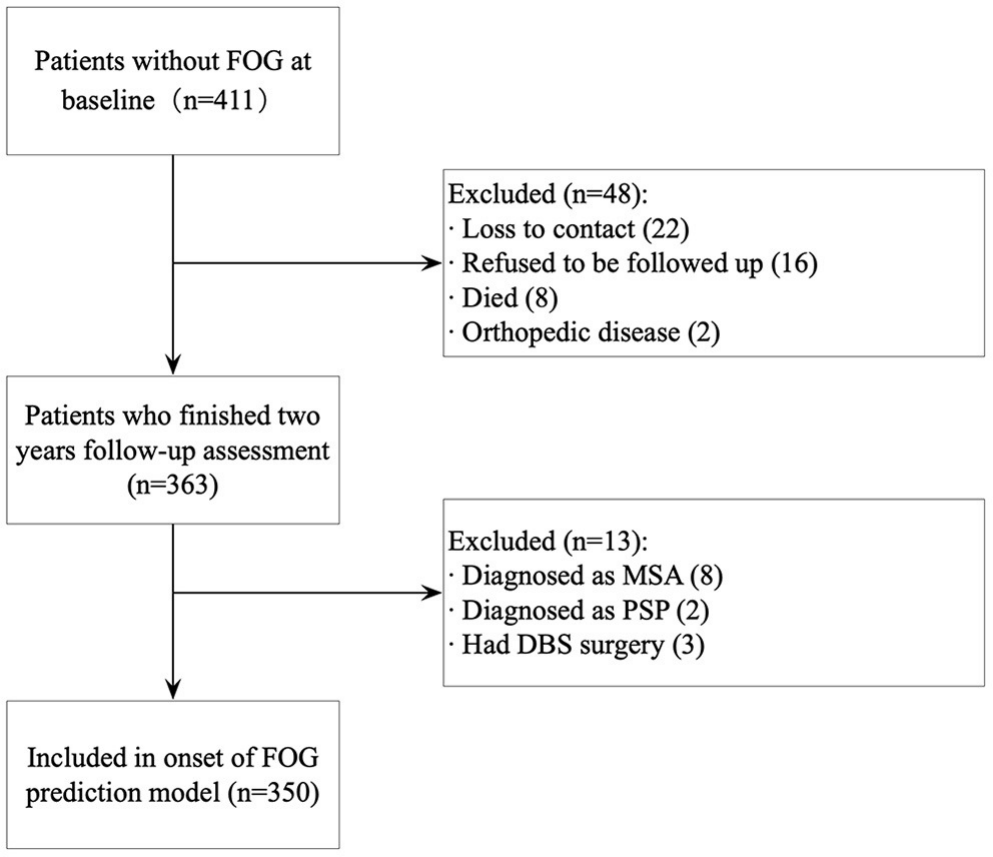
Participant selection for model development. FOG, freezing of gait; MSA, multiple system atrophy; PSP, progressive supranuclear palsy; DBS, deep brain stimulation.

A total of 350 patients with PD, who finished 2 years (20–29 months from baseline) follow-up assessment, were included in the onset of FOG prediction model development dataset. Figure 1 shows the details of patient selection and missing data for the model. The mean age of the cohort was 66.02 ± 8.52 years, 52.60% were males, and the mean disease duration was 4.41 ± 3.72 years at baseline. The mean mH&Y stage was 2.10 ± 0.78 (stage 1–1.5, *n* = 126; stage 2–2.5, *n* =151; and stage ≥3, *n* = 73). The mean HAMA and the HAMD scores were 7.48 ± 6.38 and 10.28 ± 8.52, respectively. Of the total cohort, 164 patients (46.90%) had no depressive symptoms, 140 patients (40.00%) had a mild depressive symptom, 41 patients (11.70%) had a moderate depressive symptom, and five patients (1.40%) had a severe depressive symptom.

At the follow-up assessment, 218 participants (62.30%) remained nFOG, while 132 patients (37.70%) reported that they had converted to the FOG group (NFOG-Q item 1 = 1). There are 36 (27.27%) converters and 54 (24.77%) non-converters that received a face-to-face assessment. The likelihood of being characterized as a converter or non-converter did not differ based on whether follow-up was performed remotely or in a face-to-face assessment (chi-squared test = 0.269, *p* = 0.603). Table 1 shows the baseline characteristics of the non-FOG group and conversion into the FOG group.

**Table 1:** Clinical characteristics of the study population at baseline.

	**non-FOG** **(***n*** = 218)**	**conversion into FOG** **(***n*** = 132)**	***P*** **-value^a^**
Gender, male (%)	111 (50.90)	73 (50.30)	0.426
Age (years)	65.96 ± 8.55	66.13 ± 8.49	0.857
Disease duration (years)	3.22 ± 2.77	6.37 ± 4.23	**<0.001**
mH&Y stage (%)			**0.005**
1–1.5	91 (41.70)	35 (26.50)	
2–2.5	91 (41.70)	60 (45.50)	
≥3	36 (16.50)	37 (28.00)	
UPDRS part III	20.36 ± 10.79	24.50 ± 14.22	**0.004**
TD score	5.07 ± 3.82	5.84 ± 4.84	0.120
PIGD score	2.84 ± 2.02	3.98 ± 2.83	**<0.001**
MoCA scores	22.88 ± 5.31	22.79 ± 6.18	0.877
HAMA scores	6.48 ± 5.37	9.12 ± 7.51	**0.001**
HAMD scores	8.80 ± 7.43	12.70 ± 9.59	**<0.001**
Berg balance scores	50.75 ± 7.78	48.82 ± 9.00	**0.035**
LEDD (100 mg/d)	2.90 ± 1.68	5.06 ± 2.72	**<0.001**

a *Independent sample t-test, the Mann–Whitney U test, or the chi-squared test for comparisons. Statistically significant p-values (p < 0.05) are highlighted in bold*.

*mH&Y stage, modified Hoehn and Yahr stage; UPDRS, Unified Parkinson's Disease Rating Scale; TD, tremor dominant; PIGD, postural instability and gait difficulty; MoCA, Montreal Cognitive Assessment; HAMA, Hamilton Anxiety Rating Scale; HAMD, Hamilton Depression Rating Scale; LEDD, levodopa equivalent daily dose*.

### Model Development

The backward selection process was repeated in each individual bootstrap sample (*n* = 1,000). Based on recommendations in the literature, we chose to include variables in the final model when they were selected after stepwise backward selection in more than 50% bootstrap samples (Supplementary Table 1) (27). It turns out that a longer disease duration [odds ratio (OR) = 1.214, *p* = 0.008], a higher grade of depressive symptom (OR = 1.907, *p* = 0.028), and a higher total LEDD (OR = 1.440, *p* < 0.001) were associated with the onset of future FOG. The predictor variables and their corresponding regression coefficients in the onset of FOG prediction model are shown in Table 2. The final model produced acceptable discrimination and calibration, with a C-statistic of 0.820 (95% CI: 0.771–0.865), Brier score of 0.167, and acceptable goodness of fit (Hosmer–Lemeshow chi-squared = 13.510, 8 degrees of freedom, *p* = 0.182) (Table 3).

**Table 2:** Regression coefficients of the final model.

**Outcome: Onset of FOG**	**B (SE)**	**Odds ratio**	**95% CI of Odds ratio**	***P*** **-Value**
Disease duration (years)	0.194 (0.072)	1.214	1.062–1.389	0.008
Grade of depressive symptom	0.646 (0.329)	1.907	1.085–3.352	0.028
levodopa equivalent daily dose (100 mg)	0.365 (0.111)	1.440	1.179–1.759	<0.001

*Grade of depressive symptom [1–4; none, mild, moderate, and severe based on the Hamilton Depression Rating Scale (HAMD) scores]. The three predictors appeared in more than 50% of the multivariable models generated in the different bootstrap samples (see Supplementary Table 1 for more details). Regression coefficients and SEs were averaged using Rubin's rules (23)*.

**Table 3:** Model performance parameters in the total cohort and after 5-fold cross-validation.

		**Model discrimination**	**Model calibration**		
**Validation stage**		**C-statistic^a^ (95% CI)**	**Slope^b^**	**Intercept^b^**	**Hosmer-Lemeshow test**
	Apparent performance^c^ (*n* = 350)	0.820 (0.771–0.865)	1.000	0.000	*P* = 0.182
Internal validation	Optimism corrected performance (*n* = 350)	0.792 (0.743–0.837)^d^	0.968	−0.022	
Five-fold cross-validation	Fold 1 (*n* = 70)	0.839 (0.737-0.941)	0.950	−0.227	*P* = 0.682
	Fold 2 (*n* = 70)	0.687 (0.543-0.831)	0.698	−0.066	*P* = 0.090
	Fold 3 (*n* = 70)	0.678 (0.790-0.902)	0.836	0.374	*P* = 0.421
	Fold 4 (*n* = 70)	0.763 (0.647-0.879)	0.936	−0.026	*P* = 0.310
	Fold 5 (*n* = 70)	0.801 (0.681-0.921)	1.240	−0.047	*P* = 0.552

a *C-statistic of 0.50 represents no discrimination and 1.00 represents perfect discrimination*.

b *Intercept of 0 and slope of 1 represent perfect calibration*.

c *Refers to performance estimated directly from bootstrap sample that was used to develop prediction model. Since these parameters are generated in 1,000 bootstrap samples, for each parameter the median is shown*.

d *Average optimism = 0.028 determined by internal validation in bootstrap samples (1,000 samples with replacement)*.

### Model Validation

**Figure 2:**
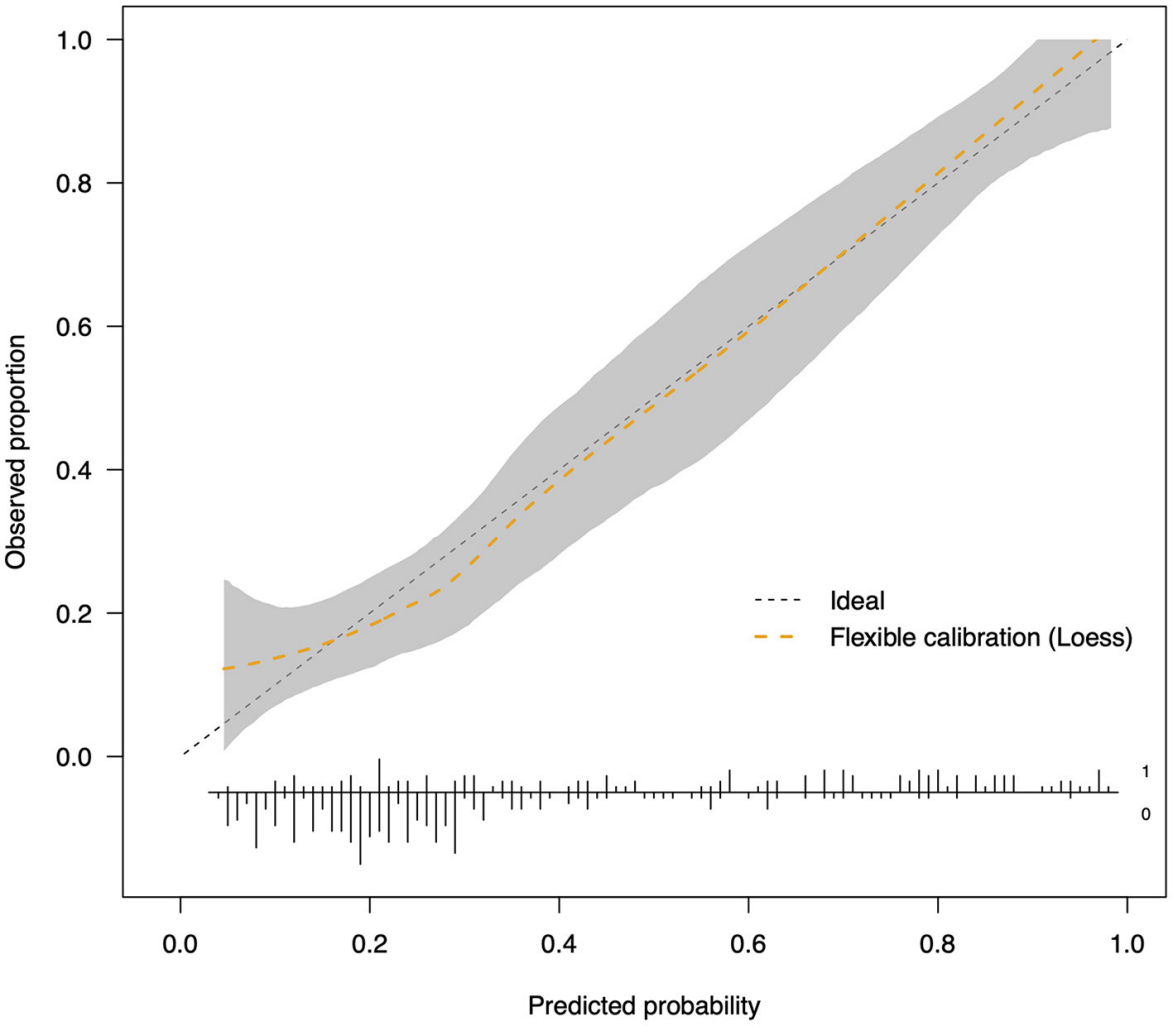
Calibration plot after internal validation by bootstrapping (*n* = 1,000). The dotted straight reference line corresponds to prefect calibration with a calibration slope of 1. When the calibration curve is above the reference line, the probabilities of freezing development are underestimated; when it is beneath the reference line, the probabilities are overestimated. The range shaded in gray represents 95% CIs of the LOESS curve. The distribution of predicted risk is shown at the bottom of the plot.

**Figure 3:**
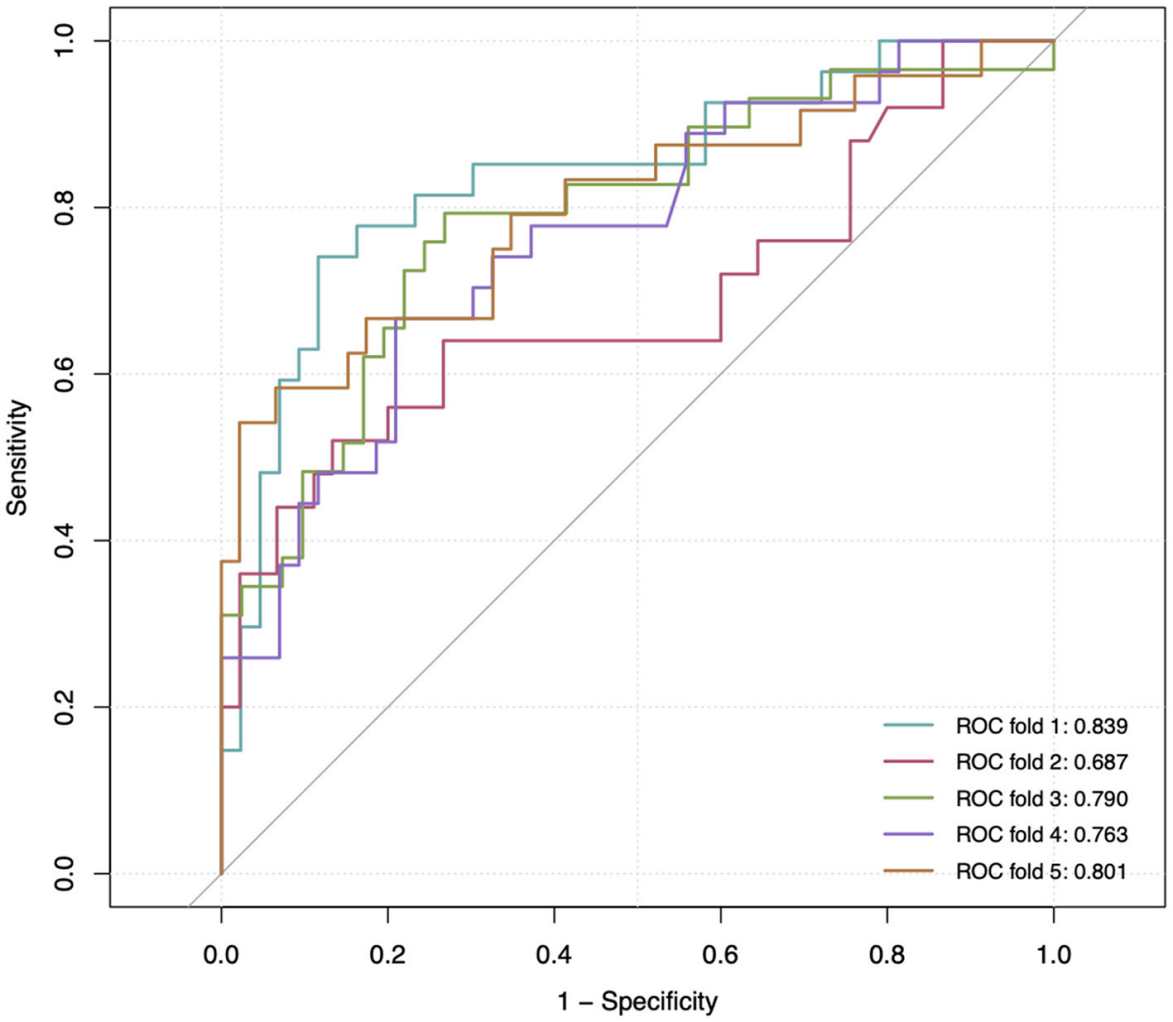
Receiver operating characteristic (ROC) curve at 5-fold cross-validation. The gray reference line corresponds to a C-statistic of 0.50, indicating a non-informative model. The area under the curve of each ROC curve is shown at the lower right corner.

Parameters of model performance are shown in Table 3. Bootstrap resampling showed negligible model optimism (average optimism = 0.028). The models had internally validated (Figure 2). Twelve significant predictors resulting from the model development were used in the 5-fold cross-validation. Rotating the 5-fold cross-validation across the five subsets, performance of the developed models remained stable with a C-statistic ranging from 0.687 to 0.839 (Figure 3). Calibration in the large was overall acceptable with calibration intercepts close to zero (ranging from −0.227 to 0.374) and calibration slopes close to one (ranging from 0.698 to 1.240) across all the five subsets. The calibration curve indicated a slight underfitting when the fold 5 subsets was the validation sample with a slope of 1.240 (Table 3).

### Model Presentation

**Figure 4:**
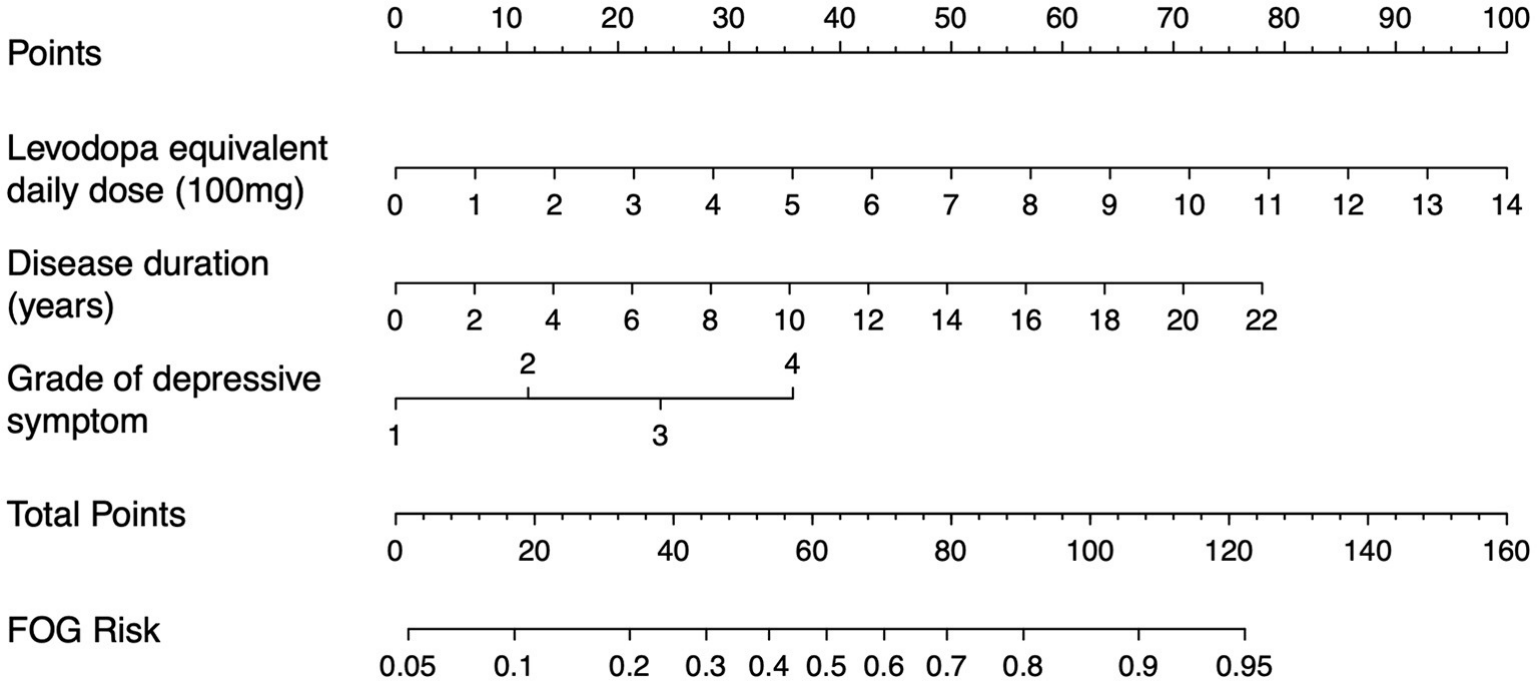
Nomogram for the prediction of 2-year onset of FOG risk. The 2-year onset of FOG risk is calculated by taking the sum of the risk points. For each factor, the number of associated risk points can be determined by drawing a vertical line straight up from the corresponding value of factor to the axis with risk points (0–100). The total point axis (0–160) is the sum of the corresponding values of factor determined by characteristic of every individual patient. Draw a line straight down from the total point axis to find the 2-year onset of FOG risk. Grade of depressive symptom [1–4; none, mild, moderate, and severe based on the Hamilton Depression Rating Scale (HAMD) scores]. For example, patient with Parkinson's disease with a 6-year disease duration, 700 mg levodopa equivalent daily dose, and moderate depressive symptom may reach 94 total points, thus referring to an 82% 2-year onset of FOG risk.

The nomogram represents a graphical tool for estimating the onset of FOG incidence at 2 years based on our model (Figure 4). In the nomogram, the categories of each factor are assigned a score using the topmost “points” scale and then all the scores are summed up to obtain the “total points” which relate to the risk of FOG. The optimal cutoff value of the Nomo score was 0.298. The specificity and sensitivity of differentiating the presence or absence of FOG were 73.85 (95% CI: 67.49–79.56) and 75.76% (95% CI: 67.53–82.79), respectively. The performance of the nomogram (including optimal cutoff, sensitivity, specificity, positive predictive value, negative predictive value, likelihood ratio, and accuracy) is shown in Table 4. These parameters are derived from the confusion matrix and can reflect the gap between the predicted and true values. The positive predictive value indicates the probability that FOG actually develops when the model predicts it will develop. The positive likelihood ratio is the ratio between the probability of a positive model prediction given development of the FOG and the probability of a positive model prediction given absence of the FOG. The negative predictive value and negative likelihood ratio represent the opposite. For example, a 68-year-old man with a 6-year history of PD, 700 mg LEDD, and moderate depressive symptoms may reach 94 total points, thus referring to an 82% 2-year onset of FOG risk. Although, he has an 82% chance to become a freezer in the next 2 years according to the model, yet, this can only be said with a 58 to 69% certainty.

**Table 4:** Performance of the prediction Nomo score for estimating the risk of FOG.

**Performance measures**	**Values**	**95% confidence interval**
Cutoff value	0.298	
Sensitivity (%)	75.76	67.53–82.79
Specificity (%)	73.85	67.49–79.56
PPV (%)	63.69	57.91–69.11
NPV (%)	83.42	78.65–87.30
LR+	2.9	2.27–3.69
LR-	0.33	0.24–0.45
Accuracy (%)	74.57	69.67–79.05

*PPV, positive predictive value; NPV, negative predictive value; LR+, positive likelihood ratio; LR-, negative likelihood ratio; FOG, freezing of gait*.

## Discussion

In this relatively large-scale, multicenter, longitudinal study, we developed a simple model that combines clinical features measured at baseline to give predictions (C-statistic = 0.820, 95% CI: 0.771–0.865) concerning the onset of FOG over the next 2 years. The multifactorial prediction model performed well in the development dataset, showed acceptable discrimination and calibration in internal validation, and remained stable in 5-fold cross-validation. The HAMD can be administered by clinicians easily without specialist equipment. Thus, this predictive model is easily translatable to the clinic.

Many cross-sectional studies identified the risk factors for FOG, but their clinical significance is limited by the inability to identify the temporal relationship between the factors involved and the onset of FOG. Several longitudinal follow-up studies have provided valuable insights into predicting freezing of gait (4–8). However, none of them provided an operable predictive model that can be used by clinicians on a daily basis. The primary purpose of this study was to develop a model that could identify high-risk individuals for future FOG onset before the FOG occurred based on an easy-to-use Nomo score. Attention to high-risk groups is beneficial for the early identification of FOG. Many freezers could benefit from optimizing the regimen of anti-Parkinson's drugs or some behavioral interventions such as cueing if they realize the presence of FOG and turn to a movement disorders specialist (28). It provided a valuable opportunity for early therapeutic measures and interventions to delay or even prevent the emergence of FOG.

In this study, patients with longer disease duration, higher LEDD of dopaminergic medications, and those with higher severity of depressive symptoms (as assessed on the HAMA) were more likely to convert to FOG than those with less disease duration, levodopa dosage, and depression scores, in line with previous reports of these characteristics being predictors of FOG in PD (7, 10, 12, 17, 29). Disease duration is one of the widely recognized risk factors of FOG. Anxiety and depression were also described as potential longitudinal predictors for future FOG (6, 7, 10–12). These two affective disorders frequently accompany PD throughout PD (30, 31). Our findings demonstrated that depression was more closely associated with the future onset of FOG, while sex and age were not associated with the development of FOG according to this study, which was confirmed by other prospective studies (7, 12, 17, 29, 32). Previous studies have shown cognition also be an important FOG predictor (5, 8, 11). After all, the pathogenesis of FOG may be closely related to cognitive function. In particular, conflict resolution deficit in the executive function domain (33, 34). However, we cannot conclude a relationship between FOG and cognition. Possible reasons for this result are that certain specific cognitive domains such as visuospatial or executive abilities are more relevant to the development of FOG. However, this study only considered global cognitive function as the total score from the MoCA. In addition, differences in assessment instruments, sample size, genetic background, and follow-up intervals may also contribute to such discrepancies.

The relationship between FOG and dopaminergic medication is complicated and confusing. We found that higher LEDD at baseline was a significant predictor of new-onset FOG during follow-up in a dose-dependent fashion. For a 100-mg increase in LEDD at baseline, the risk of incident FOG during 2-year increased by 44.0%. This finding was consistent with a long term longitudinal study (17). A possible explanation is that dopaminergic treatment increases the risk of developing FOG in the future. There is a curious phenomenon with respect to the effect of levodopa medication on FOG: FOG is generally responsive to dopaminergic medication, at least in the most common dopamine-responsive phenotype patients. But, some studies suggested that long-term pulsatile levodopa treatment may contribute to the development of FOG (35–37). Nonnekes et al. tried to explain this levodopa paradox by providing a new framework (38). Since levodopa-induced aberrant neuroplasticity is more pronounced in the substantia nigra pars compacta area (motor loops) than the ventral tegmental area (cognitive and limbic loops) due to greater dopaminergic denervation (39), levodopa treatment leads to a growing mismatch between activated cognitive and limbic loops (causing a desire to walk), but understimulated motor loops (causing an inability to initiate a desired step). This dissociation between desire and capacity may result in FOG. However, the evidence for levodopa causing FOG is very weak. In the meantime, it is also important to note that overall life expectancy, as well as other medical care, has increased. Thus, it may also be that part of the higher FOG progression seen since levodopa introduction is due to other reasons. More prospective trials with long follow-up comparing patients with early levodopa treatment vs. delayed levodopa treatment were needed to clarify these complex relationships. Although several studies have found that neuroimaging or CSF markers were associated with the development of FOG (5, 8, 9). These measures were not included in this study for the sake of model practicability.

The strengths of this study include that it is built from easily available clinical and demographic variables, implying that it can be straightforwardly applied in clinical practice and is readily amenable to further external validation in many other cohorts that have routine data available for such a purpose. The assessment of outcome measures, including demonstrating typical FOG phenomenon to the patients, their family members, or their caregivers by an experienced clinician, is relatively accurate. The low prevalence of the FOG in the consultation room most likely relates to participants being assessed in the “on” state when their mobility was optimal, whereas the NFOG-Q item reflects a 1-month time period that would have included the end of levodopa dose motor fluctuations. Thus, this self-reported questionnaire is good at detecting FOG onset as long as the patients are able to identify those phenomena well. Accurate assessment of outcome events is critical to the development of clinical prediction models.

Nonetheless, this study also has several limitations. First, the lack of an independent external validation cohort makes the external generalizability of the model unknown. Second, the interval between follow-up visits among patients was not strictly restricted. More number of visit points with a shorter regular time interval could help to determine the exact conversion timepoint. It is worth considering this issue in the future study design. Third, we did not discriminate the predictors of FOG in patients with different medication states (“ON” or “OFF”), so the role of drug therapy in the pathogenesis of FOG was hard to explain (40). Fourth, no FOG-specific elicitating tasks have been included in the assessments, which could have improved the objective FOG detection. Fifth, since DBS is in some cases considered for FOG treatment (41) and may otherwise lead to interference with the judgment of whether converted to FOG. Three patients who had DBS surgery during follow-up assessment were also excluded. The effectiveness of DBS in the treatment of FOG remains controversial to date (42). Clarifying the relationship between DBS and FOG development with more samples of patients with DBS can be worthwhile investigating in the future.

In conclusion, we have developed and cross-validated a risk prediction model for the future onset of FOG based on three easy-to-measure variables. The model is reasonably calibrated and prediction accuracy is acceptable. The clinical utility assessment of this model showed potential for improved risk counseling, although the remedy of FOG remains challenging.

## Data Availability Statement

The raw data supporting the conclusions of this article will be made available by the authors, without undue reservation.

## Ethics Statement

The studies involving human participants were reviewed and approved by the Ethics Committee of Xinhua Hospital Affiliated to the Shanghai Jiao Tong University School of Medicine. The patients/participants provided their written informed consent to participate in this study.

## Author Contributions

JZ and YW contribute to the collecting clinical data, statistical analysis, and drafting the manuscript. LS, NW, and ZZ contribute to the recruitment of subjects and gathering clinical data. ZL and JG contribute to the conceptualization, critical revision of the manuscript, and study supervision. All authors contributed to the article and approved the submitted version.

## Funding

This study was supported by the National Key R&D Program of China (2017YFC1310300), the Projects of National Natural Science Foundation of China (81974173, 81771211, and 81703852), the Projects of the Shanghai Committee of Science and Technology (17401901000 and 19401932100), the Innovation Research Team of High-level Local Universities in Shanghai, Shanghai Municipal Commission of health (2019SY024), and the Special Project of Integrated Traditional Chinese and Western Medicine in Shanghai General Hospital (ZHYY-ZXYJHZX-202021).

## Conflict of Interest

The authors declare that the research was conducted in the absence of any commercial or financial relationships that could be construed as a potential conflict of interest.

## Publisher's Note

All claims expressed in this article are solely those of the authors and do not necessarily represent those of their affiliated organizations, or those of the publisher, the editors and the reviewers. Any product that may be evaluated in this article, or claim that may be made by its manufacturer, is not guaranteed or endorsed by the publisher.
