# Supplementary material for: Longitudinal Prediction of Freezing of Gait in Parkinson's Disease: A Prospective Cohort Study
Source: Front Neurol. 2022 Jan 3;12:758580. doi: 10.3389/fneur.2021.758580 (PMC8761770; doi:10.3389/fneur.2021.758580)
Supplement: Supplementary file 1 [file Table_1.DOCX]

**Supplementary Table**

Supplementary Table 1 shows the full set of candidate predictors. The backward selection process was repeated in each individual bootstrap sample (n=1000). Based on recommendations in the literature, we chose to include variables in the final model when they were selected after stepwise backward selection in more than 50% bootstrap samples.

|  | bootstrap inclusion frequency (%) |
| --- | --- |
| Gender | 0 |
| Age (years) | 38.0 |
| Disease duration (years) | 99.7 |
| mH&Y stage | 30.7 |
| UPDRS part Ⅲ | 32.4 |
| TD score | 37.9 |
| PIGD score | 44.8 |
| MoCA scores | 46.4 |
| HAMA scores | 43.8 |
| HAMD scores | 66.4 |
| Berg balance scores | 28.0 |
| LEDD (100mg/d) | 100 |
